# Diurnal retinal and choroidal gene expression patterns support a role for circadian biology in myopia pathogenesis

**DOI:** 10.1038/s41598-023-50684-2

**Published:** 2024-01-04

**Authors:** Richard A. Stone, John W. Tobias, Wenjie Wei, Jonathan Schug, Xia Wang, Lixin Zhang, P. Michael Iuvone, Debora L. Nickla

**Affiliations:** 1 Department of Ophthalmology, Perelman School of Medicine, University of Pennsylvania, Philadelphia, PA USA; 2 Penn Genomics and Sequencing Core, Perelman School of Medicine, University of Pennsylvania, Philadelphia, PA USA; 3 Department of Biomedical Sciences and Disease, New England College of Optometry, Boston, MA USA; 4 Department of Ophthalmology and Department of Pharmacology and Chemical Biology, Emory University School of Medicine, Atlanta, GA USA

**Keywords:** Genetics, Neuroscience, Medical research, Pathogenesis

## Abstract

The prevalence of myopia (nearsightedness) is increasing to alarming levels, but its etiology remains poorly understood. Because both laboratory and clinical findings suggest an etiologic role for circadian rhythms in myopia development, we assayed gene expression by RNA-Seq in retina and choroid at the onset of unilateral experimental myopia in chick, isolating tissues every 4 h during a single 24-h period from myopic and contralateral control eyes. Occluded versus open eye gene expression differences varied considerably over the 24-h sampling period, with some occurring at multiple times of day but with others showing differences at only a single investigated timepoint. Some of the genes identified in retina or choroid of chick myopia were previously identified as candidate genes for common human myopia. Like differentially expressed genes, pathways identified by Gene Set Enrichment Analysis also varied dramatically by sampling time. Considered with other laboratory data, human genetic and epidemiology data, these findings further implicate circadian events in myopia pathogenesis. The present results emphasize a need to include time of day in mechanistic studies of myopia and to assess circadian biology directly in trying to understand better the origin of myopia and to develop more effective therapies.

## Introduction

Myopia (nearsightedness) develops from a mismatch between the optical properties of the tissues in the front part of the eye and the length of the vitreous chamber, such that distant images focus anterior to the retinal photoreceptors^1^. Most commonly, myopia results from an elongated eye. Besides the functional inconvenience of blurred vision, myopia predisposes to many blinding diseases in adulthood, including various macula and retinal degenerations, retinal detachments, glaucoma and certain forms of cataract^2^. Myopia accordingly is a significant risk factor for acquired blindness in adults. None of the many available optical and surgical approaches to improve the defocused vision are known to reduce the development of myopia-associated ocular disease. Particularly worrisome, the prevalence of myopia is increasing dramatically worldwide, reaching a prevalence in young adults of some 80–90% in regions of East and Southeast Asia and up to 40–50% in the United States and Europe^3–5^. It is estimated that some 50% of the world's population may be myopic by 2050^6^. Why myopia develops and why its prevalence is increasing remain unclear despite over a century of clinical and basic investigations, speculations and hypotheses.

Research in experimental animals, with confirmatory evidence in children, has demonstrated that visual input governs refractive development^7–10^. For example, wearing an image-degrading diffuser induces ipsilateral form deprivation myopia as frequently studied in chicks and young mammals^11^. From this and other approaches, much research has identified the retina as governing ocular growth and myopia. Visual input also modulates the thickness of the choroid, the tissue underlying the retina. The choroid is hypothesized to interact with the retina and with the sclera in a signaling cascade that regulates ocular growth and refraction^12–14^. Laboratory methods applied to understand myopia pathogenesis have revealed an overwhelming number of signaling molecules, enzymes, transcription factors and pathways that may impact ocular growth and refraction^9^. Besides blur or other image qualities, the intensity and color of ambient light impact refractive development in experimental animals and likely in humans^15–17^.

Developing a unified framework to understand the pathogenesis of clinical myopia has proved challenging. Emerging from contemporary laboratory studies in animal models and clinical research is the notion that refractive errors may arise from circadian disruption^9,18–20^. The extent of these observations, detailed below, reinforces a key role for circadian rhythms in regulating ocular development and suggests that circadian biology may lead to a much-needed framework to understand myopia pathogenesis.

Despite suggestions of circadian impact^9^, only some molecular studies of the retina have controlled for or precisely reported time of day, as illustrated for chick^21–25^. Also considering the choroid’s likely interactions with retina in governing eye growth^14^, very few molecular investigations have separately included choroid, and none have examined gene expression in either tissue across a full day. Here, we studied gene expression in the retina and separately in the choroid at the initiation of form-deprivation myopia in chick, a widely studied experimental myopia model^26^. For choroid, we are aware of only sparse prior data contrasting gene expression in occluded versus open eyes^21^. In comparing eyes developing myopia with contralateral control eyes in each tissue, we discovered striking differences throughout the day of altered genes. A limited number of differentially expressed chick genes overlapped with genes implicated in human myopia. Biological pathways in both tissues, like individual genes, vary with sampling times throughout the day. These results not only further buttress a role for circadian biology but also reveal time of day as a novel parameter for including in studies addressing myopia mechanisms.

## Results

As expected for the full dataset, tissue (retina vs. choroid) was the strongest factor separating gene expression results in the samples. A secondary grouping, not corresponding to any experimental design parameters, separated the samples by sex as identified by the presence/absence of genes located on the W sex chromosome of female birds. Sex was included in statistical models going forward. Overall, retina samples were less variable than choroid samples. Parallel analyses addressed retina and choroid samples separately, examining the effects of eye and sampling time.

### The expression levels of many genes vary over time

For either retina or choroid, the expression levels of some 50–60% genes varied over the 24-h day (Table 1; Suppl. Tables S1A–S1D). Because we did not study constant light or dark conditions, diurnal or circadian effects cannot be distinguished from acute light effects. Because we tested only one daily cycle, the gene expression patterns here are properly described as diurnal rather than circadian^27^.

**Table 1:** Number of genes with expression levels varying over a 24-h day, using the criterion of p-adj < 0.05.

Eye	Retina		Choroid	
	Number of genes^a^	%^b^	Number of genes^a^	%^b^
Occluded eye	9930	57.9	9851	57.5
Open eye	8536	49.8	10,421	60.8

See Suppl. Tables S1A–S1D for complete lists in each tissue of all identified genes, sorted by the p-adj values for the variability of each.

^a^Number of varying genes, identified by Ensembl gene id’s, in each tissue/eye with p-adj < 0.05.

^b^Based on 17,136 total chicken Ensembl gene identifications.

**Figure 1:**
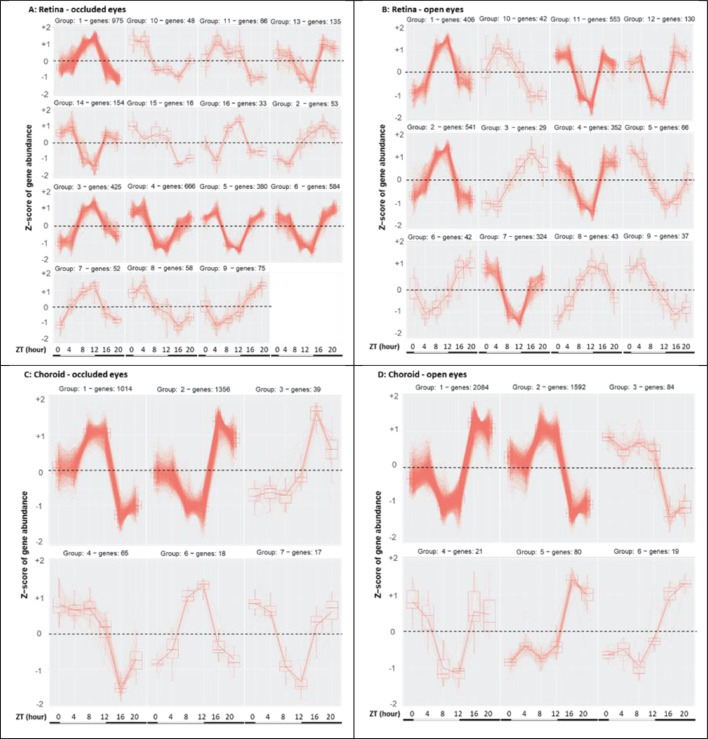
Patterns of variable gene expression in retina and choroid. The variable patterns of gene expression over 24 h are shown for the occluded eyes (**A**) and contralateral open eyes (**B**) of retina and for the occluded eyes (**C**) and contralateral open eyes (**D**) of choroid. These clustered patterns of expression were generated from a subset of varying genes using the statistical criteria described in the text. The density of each tracing represents the number of genes conforming to a specific pattern. The bottom bars on each panel illustrate the light:dark phases, with the light phase (white bar) beginning at ZT0, and the dark phase (black bar) beginning at ZT12. Abscissa: the sampling times in ZT (h). Ordinate or Z-scores: 0 = mean, with non-zero values corresponding to ± S.D.

To group these diurnal variations into patterns, we clustered genes with variable expressions into discrete expression patterns over 24 h for each tissue and eye. To reduce the large number of varying genes into more manageable numbers, the clustering model for retina used a p-adj cutoff of 1 × e^−8^ (n = 3745 genes for open eyes; n = 2584 genes for occluded eyes); for the clustering model for choroid, a p-adj cutoff of 1 × e^−11^ (n = 2522 genes for open eyes; n = 3895 genes for occluded eyes). For retina, the gene expressions of occluded eyes clustered into 15 patterns; those of open eyes, into 12 patterns (Fig. 1). For choroid, the gene expressions of occluded eyes clustered into 6 patterns; those of open eyes, into 6 patterns (Fig. 1). In each tissue and eye, these patterns demonstrated the complexity of changing gene expression levels throughout the day. Many, though not all, varying genes showed highest or lowest expression levels near the end of the light phase.

### Gene expression differences between occluded and open eyes vary over time

In studying mechanisms for monocular experimental myopias, tissue levels of gene expression, neurotransmitters or proteins are typically compared between occluded and contralateral control eyes^9,18^. Using the statistical criterion of p-adj < 0.05 for both retina and choroid, the differences in gene expression between occluded and contralateral open control eyes varied markedly by time of tissue sampling (Table 2; Suppl. Tables S2A, S2B). Except for ZT 04, the number of differentially expressed genes was greater in choroid than in retina. For each tissue, the number of differentially expressed genes increased between ZT 0 (lights on) and ZT 8 before reducing in number.

**Table 2:** Numbers of genes with occluded versus open eyes expression differences at each time, using the criterion of p-adj < 0.05.

Sampling time, ZT in hours	Retina					Choroid				
	Number of retinal genes	Direction of gene expression changes in occluded eye relative to open eye; number and %				Number of choroidal genes	Direction of gene expression change in occluded eye relative to open eye; number and %			
		Up		Down			Up		Down	
0	28	4	14.3%	24	85.7%	39	24	61.5%	15	38.5%
4	62	10	16.1%	52	83.9%	27	9	33.3%	18	66.7%
8	119	53	44.5%	66	55.5%	673	205	30.5%	468	69.5%
12	21	5	23.8%	16	76.2%	51	23	45.1%	28	54.9%
16	4	0	0.0%	4	100.0%	96	64	66.7%	32	33.3%
20	0	0	0.0%	0	0.0%	193	156	80.8%	37	19.2%
Overall	24	3	12.5%	21	87.5%	32	20	62.5%	12	37.5%

Genes are listed with expression differences between occluded and open eyes that met the criterion of p-adj < 0.05.

“UP” = occluded/open eyes: + fold-change; “DOWN’ = occluded/open eyes: − fold-change.

“overall” = those genes with a response to occlusion (i.e., form-deprivation) that differed statistically between occluded versus contralateral open eyes over the 24-h day (see text).

See Suppl. Tables S2A and S2B for the specific genes meeting the p-adj < 0.05 criterion for each tissue, time and condition, ranked by log_2_ fold change.

For retina, gene expression differences occurred mostly from ZT 0 through the light phase, peaking at ZT 8. By ZT 16, only four genes were differently expressed; at ZT 20, no gene expression differences were detected between the retinas of the two eyes. Most of these genes were downregulated in the occluded eye relative to its contralateral eye. A marked reduction in the number of differentially expressed genes in retina occurred during the dark phase.

The choroid demonstrated a greater number of differentially expressed genes between occluded and open eyes than retina, particularly at ZT 8, ZT 16 and ZT 20 (Table 2). Choroidal inter-eye differences in gene expression also varied by time, but not with the marked reduction in the number of differentially expressed genes during the dark phase as in retina. The patterns of up- and downregulated genes also were more complicated. A majority of the affected choroidal genes were upregulated in occluded eyes during the dark phase (ZT 16 and ZT 20) and at the transition to light (ZT 0); during and at the end of the light phase (ZT 4, ZT 8 and ZT 12), a majority of the affected choroidal genes were downregulated in occluded eyes (Table 2).

For both retina and choroid, the “overall” category derived from analyses that considered all replicates at all time points simultaneously and prioritized genes with a response to occluder wear that was similar in magnitude and direction at all time points. This category identified a small number of genes that differed between occluded and open eyes over the full 24-h period. There was no requirement that the genes in the overall category would significantly differ between occluded and open eyes at each time point individually (Tables 2, 3; Suppl. Tables S2A, S2B).

**Table 3:** Genes with occluded versus open eye differences over the full 24-h period.

Retina		Choroid		
NOG	ARID5B	PLLP	GJC2	IGF2
G0S2	LONRF3	MBP	NGF	CDON
PRDM1	NADK	PTHLH	PPP4R4	HTR1B
PWP1	LONRF1	CORIN	CLEC3B	HTR2B
MAPK4	RAD54L2	KRT7	SLMAP	GAS1
GABRR2	PCSK1	BMP3	SMURF1	PTX3
SPRED1	CALCA	GRM3	PDGFD	CER1
CMIP	DUSP4	ASB2	C1orf198	TNNT1
C1orf21	UTS2B	VGLL3	FUT11	MYL3
PDP2	BMP2	C1QTNF7		
TMEM196				

Named genes from the “overall” category of Table 2 with differences between occluded and open eyes over time, p-adj < 0.05. See Suppl. Tables S2A and S2B for more information on specific genes, including the non-named genes in this category that are not shown here.

### Interactions of occluded versus open eye with time occurred for a limited number of genes

**Figure 2:**
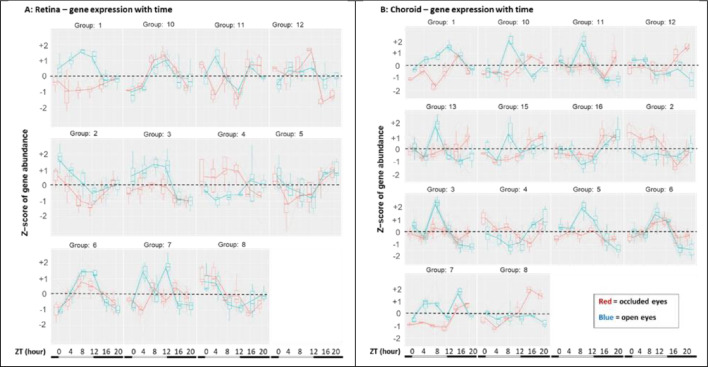
Gene expression patterns where occluded and open eye differences interact with time. The patterns over 24 h are shown for the interaction of time with gene expression in the occluded versus open eyes for retina (**A**) and for choroid (**B**). Because of the limited number of genes meeting the criterion of p-adj < 0.05 or p-adj < 0.1, these clusters were generated from genes with looser statistical criteria (see text). The individual genes with occlVopen * time interactions meeting the statistical criterion of p-adj < 0.10 are listed in Table 4 and Suppl. Tables S3A, S3B and are shown in the heatmaps of Suppl. Figs. S1A, S1B. The bottom bars on each panel illustrate the light:dark phases, with the light phase (white bar) beginning at ZT0, and the dark phase (black bar) beginning at ZT12.Abscissa: the sampling times in ZT (h). Ordinate or Z-scores: 0 = mean, with non-zero values corresponding ± S.D.

For each tissue, we modeled the interaction of occluded versus open eyes with time (“occlVopen* t ime”) to identify genes where the expression pattern beneath a diffuser varied over 24 h in a pattern statistically different from that of the contralateral open eye. However, the number of genes meeting either the p-adj < 0.05 criterion (retina, n = 21; choroid, n = 36) or the p-adj < 0.1 criterion (retina, n = 31; choroid, n = 71) was too few to meaningfully model the patterns of gene expression over time into clusters. Accordingly, we arbitrarily selected a p-adj of 0.4 for retina and 0.2 for choroid that yielded 82 retinal genes and 160 choroidal genes and clustered the interaction patterns (Fig. 2). Depending on the gene cluster, specific gene expressions in occluded eyes were higher or lower than the open eyes at most times or only at some times; in some clusters, the comparative gene expression levels between the two eyes even reversed during the day (e.g., Fig. 2A, group 4). Heatmaps show the diurnal fluctuation pattern of those genes meeting the occlVopen * time models with p-adj < 0.10 for retina and choroid (see Suppl. Figures S1A, S1B, Table 4, and Suppl. Tables S3A, S3B).

**Table 4:** Genes with expression differences between the occluded versus open eye interacting with time over the full 24-h period.

Retina		Choroid			
VIP	ACOX2	ATOH8	EML5	CDR2L	NOS1
DIO2	NOG	ID3	NMUR1	RNF165	CNKSR2
UNC5C	HBEGF	RGS16	RAB3A	CDC25A	GIN1
GLS2	RSPO2	KRT40	BTG2	DIS3L2	SGO1
PCSK1	NR2F2	ID2	LIX1	LECT2	CD24
NADK	PWP1	RGS8	CYR61	DEFB4A	TMEM59L
SPON1	PRDM1	LBH	INTS11	CATHL2	IGF-I
PER2	MGP	SLMAP	ETV6	CATHL1	ASTN1
UTS2B	COL9A2	TGFB3	C1orf198	LYG2	CDH8
BMP2	CRHBP	RASL11A	GPRIN2	AvBD1	CACNB3
MAFF	PDE6B	NOV	NPTXR	BD7	NCAPG2
DUSP4	G0S2	VGLL3	FXYD6	ID4	NUSAP1
TH	MFSD2A	NSMF	SAMD11	AvBD6	PISD
		ADGRB2	SNPH	TMEM100	FOXM1
		WASF1	SKIL	TBC1D9	ASTN1
			MSI1	SYT9	

Named genes with occluded versus open eyes differences that interact with time over 24 h (i.e., occlVopen * time interaction) with p-adj < 0.10. See Suppl. Tables S3A and S3B for more information on specific genes, including gene description, cluster assignments, statistical criteria, and non-named genes in this category for each tissue. See Suppl. Figs. S1A and S1B for heatmaps of the expression of these genes.

### Some genes developed occluded versus open eye expression differences at more than one time

**Figure 3:**
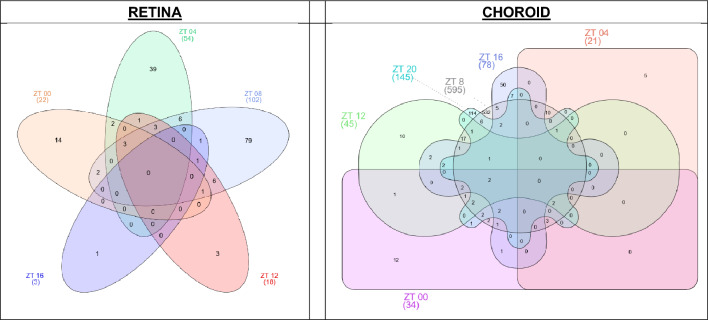
Venn diagrams to indicate the genes showing occluded versus open eye differences at specific times in each tissue. For each tissue, the Venn diagrams identify the number of genes with different expression levels in the occluded versus open eyes at more than one time, using the criteria of p-adj < 0.05 for the occluded versus open eyes (Suppl. Tables S2A and S2B). The Venn diagrams in the two panels assume different shapes because the retina developed no differentially expressed genes at ZT 20, resulting in one less time to include than in the choroidal diagram. Suppl. Tables S4A and S4B identify the times, the number of genes at each time, the specific genes identified at more than one time, the directions of gene expression change and the log_2_ fold changes.

We used Venn diagrams^28^ to identify genes with expression levels differing statistically between occluded versus open eyes at more than one time (Fig. 3). Regardless of whether many or few genes showed occluded versus open eye differences at a specific time (Table 2), a limited number of these genes developed occluded versus open eye expression differences at more than one time in either tissue (Suppl. Tables S4A, S4B). For retina, the same direction of fold change was observed for each individual gene differentially expressed at more than one time (Suppl. Table S4A). In the choroid, the fold changes of individual genes differentially expressed at more than one time usually, but not always, developed in the same direction at the distinct times (Suppl. Table S4B).

Among genes with differential expression at two times, 17 of 21 retinal genes and 45 of 63 choroidal genes were altered at consecutive times (Table 5A). Table 5B provides a listing of all genes differentially expressed at 3 or more times.

### Biochemical and signaling pathways in myopia initiation

To model further the data, we conducted Gene Set Enrichment Analysis (GSEA) to identify pathways potentially involved in initiating myopic eye growth. In doing so, we adapted the traditional approach of comparing occluded eyes to contralateral open eyes. Specifically, we identified (1) pathways in the GSEA report enriched in the occluded eyes relative to contralateral open eyes that were generated from genes with increased expression in occluded relative to the contralateral eyes; and (2) pathways enriched in the open eyes relative to contralateral occluded eyes that, viewed in terms of the traditional approach of comparing occluded to open eyes, corresponded to pathways generated from genes with decreased expression in occluded relative to contralateral open eyes. Using a conservative FDR < 0.05 for pathway identification, we assessed GSEA for each time, the overall category and the occlVopen * time interactions (see Suppl. Tables S2A, S2B, S3, S5A, S5B; Discussion).

### Overlapping chick genes and gene candidates associated with human myopia

We compared genes identified in the occluded versus open eye comparisons in form-deprivation myopia in chicks (p-adj < 0.05; Suppl. Tables S2A, S2B, S3) with an extensive list of human genes recently assembled from GWAS and linkage studies^22^. Only a limited number of differentially expressed genes in chick form-deprivation myopia associated with human myopia (Table 6). The retinal genes in chick coinciding with the human genes were identified at chick light-phase sampling times ZT 0, 4, 8 and the overall category; there was no significant overlap at ZT times 12, 16 and 20. In striking contrast to retina, the choroidal genes in chick coinciding with human genes occurred at ZT times 12 and 16—that is, the dark phase—and not at any of the times or overall category as in retina. The overlaps between the significant genes in the occlVopen * time interaction model and the human gene lists for either tissue were not statistically significant.

## Discussion

Motivating the present study, extensive evidence supports the hypotheses that circadian biology influences refractive development and that myopia may arise, at least in part, by circadian dysregulation^9,18^. In laboratory animals and humans, the anatomical dimensions of the eye and its components oscillate during the day in patterns that appear to influence refractive development^13^. Altered retinal expression of clock and circadian rhythm-related genes have been identified in experimental myopia of chick and mouse^22,23,29–31^. Specific visual alterations that experimentally induce refractive errors in chick each alter the diurnal expression of clock and circadian rhythm genes^24^. In mice, retinal-specific knockout of the clock gene *Bmal1* induces myopia^32^, knockout of the melanopsin gene in retina alters normal eye development and augments experimental myopia^33^, and ablating intrinsically photosensitive retinal ganglion cells (ipRGCs) suppresses myopia^34^.

In addition, human GWAS findings have identified hundreds of specific genes and genetic loci associated with myopia and/or refractive error, including genes that point to genetic networks involving light sensitivity and circadian control^19,20^. The scope of laboratory and clinical observations strengthens the hypothesis that circadian rhythms may impact ocular development and suggests that circadian biology may provide a basis to understand myopia pathogenesis and to develop novel therapies to normalize eye growth during childhood.

The retina is presumed to initiate the signals that regulate refractive development that then act at the choroid and subsequently at the sclera to control overall eye size and refraction^35^. We applied a vision degrading diffuser over one eye for comparison to the contralateral eye in chick, a widely studied and pertinent laboratory technique to induce ipsilateral myopia that is termed form-deprivation myopia^26,36^. The mechanisms responsible for the onset of myopia likely differ from those responsible for the progression of established myopia both in animals and in humans^37,38^. Since patterns of gene expression in many tissues change over the course of a day^39,40^, we compared retinal and choroidal gene expression in occluded and contralateral open eyes over 24 h after the first full day of monocular occlusion in chick as a model for myopia onset. Assessing myopia progression will require examining tissues after longer periods of altered visual input. Since retina and choroid each contain multiple cell types, bulk tissue assays as performed here cannot distinguish the activity of individual cell types or resolve the interactions of different cell types where gene expression changes in one cell type might augment or negate those in another cell type.

The expression levels of a great number of genes in both retina and choroid varied over 24 h (Table 1; Suppl. Tables S1A–S3D). The fluctuation patterns differed between genes, between tissues, and whether eyes were occluded or open (Fig. 1). Such marked variability between time and visual status presents challenges in selecting optimal times to study individual genes because sampling time clearly impacts gene expression levels.

A common approach to identify perturbations in biochemical or molecular mechanisms in monocular form-deprivation myopia compares occluded eyes to contralateral open control eyes. In comparing tissues from occluded to open eyes here, there were marked differences in the number, the identity and the proportion of up-regulated and down-regulated genes in retina or in choroid; all of these differences depended on time of day. Table 2 and Suppl. Tables S2A, S2B provide these data for occluded versus open eye gene expression differences meeting the p-adj < 0.05 criterion, ranked by log_2_ fold change. For retina, the between-eye gene expression differences occurred principally during the light phase, reminiscent of the light phase effects originally reported for retinal dopamine metabolism^41^. For choroid, many more genes were affected, and the gene alterations were found throughout the 24-h day. The greatest number of differentially affected genes in each tissue developed at ZT 08. Based on these data, the time of expression of individual genes presumably needs to be incorporated into experimental investigations of myopia pathogenesis; but other than the present study, available investigations do not incorporate time of day in ways that could guide future research. Complete lists of differentially expressed genes comparing occluded versus open eyes at any p-adj level, with gene names and fold changes, appear in Suppl. Tables S6A (retina) and S6B (choroid).

The variability between occluded and open eyes over the day identifies important qualifications about existing molecular reports of refractive mechanisms. Prior investigations with tissue harvested at only a single time of day would have sampled only some of the many molecular effects found here. In those studies, either casually controlling or failing to control for time of tissue harvest, mixing samples may have biased the outcome. Thus, the present results provide an important caveat to interpreting existing publications on molecular mechanisms in experimental myopia. While direct tests are needed, we suspect that a similar caveat might apply to biochemical assays of ocular tissues in experimental myopia because gene expression impacts protein products, although in complex ways.

Two analyses are particularly pertinent to the circadian rationale for this investigation: the occlVopen * time interactions and the overall analyses. In each tissue, the Occl occlVopen * time interaction model identified several genes. These interacting gene expression patterns fell into specific clusters depending upon tissue (Fig. 2, Table 4, Suppl. Table S3, Suppl. Figs. S1A, S1B). Some of those genes have been identified in previous myopia studies and/or may prove fruitful in future investigations—as a few examples: VIP^37^, DIO2^22^, and TH^41^. Diffuser wear had limited effect on the expression of clock genes in retina or choroid in our prior study^24^; here, we identified a modest biphasic effect of diffuser wear only in the retinal expression of PER2 (Suppl. Fig. S1; Suppl. Table S3A; Fig. 2A, group 2). The occlVopen * time interaction model is informed by data from many time points and provides more robust identifications than an analysis at a single time. Still, many genes were also highlighted at specific times in the present study.

In the overall analysis, only a small proportion of genes showed persisting inter-eye differences in each tissue over 24-h by statistical criteria (overall category in Tables 2, 3; Suppl. Tables S2A, S2B). A few examples of potentially informative differentially expressed genes throughout the day in either tissue also identified in other genome-wide assessments of chick include: BMP2 and/or its inhibitor NOG^22,29^, CALCA^29,37,42^, UTS2B^29,43^, BMP3^23^, GRM3^23^, NGF^29^, and HTR1B^23^. Those genes with persisting differences over the day may have particular significance for precipitating myopic eye growth, but direct investigations will be necessary to substantiate such a hypothesis.

As a consequence of the statistical modeling, some genes (e.g., BMP 2, NOG, and DUSP4) with inter-eye differences over the full 24 h (Table 3) also appear in the list of genes that have inter-eye differences interacting with time; Table 4). Irrespective of these apparent statistical contradictions, identifying these genes still supports their potential utility for future studies of myopia pathogenesis.

As another approach to assess individual genes, Venn diagrams^28^ categorized specific genes where inter-ocular differences in expression developed at more than one time (Fig. 3; Table 5A, B; Suppl. Tables S4A, S4B). The majority of genes differentially expressed at more than one time occurred at only two times in each tissue. Genes with statistically non-variable differences over the full 24-h period (Table 3) do not appear as genes with occluded versus open eye differences at all 6 times because of the nature of the statistical modelling. Perhaps genes differentially expressed at two times may exert more refractive impact than genes differently expressed at separated times or at only one time. With the 4-h gap between testing times, however, gene expression differences greatest at an intermediate time could have affected expression at two successive times and might not reflect a particularly extended effect. Genes differentially expressed at three or more times may have more mechanistic implications than those affected at one or two times. Many of the genes differentially expressed at multiple times have generated past interest or could be studied productively in the future. Some examples include: BMP2, TH, NTS^37^, GCG^23,29,43^, UTS2B^29,37,43^, DIO2^22^, VIP^37^, GAD2^23,29^, NGF^29^, MYL3^29^, and BMP3^23^. As with other approaches to addressing inter-eye differences in gene expression, direct investigation is needed to decide if the mechanistic impact of genes differentially expressed at several times differs from that of genes differentially expressed at one time.

**Table 5:** (A) Number of genes with occluded vs. open eye differences at more than one ZT time (p-adj < 0.05). (B) Specific genes with inter-eye expression differences at 3 or more times (p-adj < 0.05).

Number of ZT times that individual genes showed inter-eye differences in expression	Number of genes with inter-eye differences in expression	
	Retina	Choroid
**A**		
2	21	63
3	6	11
4	3	8
5	0	5

Number of ZT times that individual genes showed inter-eye differences in expression	Specific genes with inter-eye differences in gene expression at three or more times	
	Retina	Choroid
**B**		
3	PCSK1, DIO2, GLS2, VIP, GAD2, ENSGALG00000005011	PPP4R4, NELL2, PDGFD, HAS2, GJC2, SLMAP, VGLL3, GDPD4, MXRA8, CYGB, HTRA3
4	UTS2B, DUSP4, NOG	C1QTNF7, BMP3, ASB2, PTX3, FAM26E, HTR1B, DEPDC1, GFPT2
5	–	CER1, GRM3, CORIN, PTHLH, ENSGALG00000017029

For each tissue, Table 5A identifies the number of genes with occluded versus open eye differences at more than one time; and Table 5B lists the specific genes differentially expressed at 3 or more times in occluded versus open eyes, by the criterion of p-adj < 0.05 used for selecting individual genes/times for the Venn Diagrams. Suppl. Tables S4A and S4B provide the gene descriptions, directions of gene expression changes and the log_2_ fold changes at each ZT time, with the specific genes grouped together by common ZT times of their gene expression changes.

We used GSEA to classify the differences for occluded versus open eyes into known pathways as a tool to generate hypotheses and influence future research. Incorporating all gene expression values of occluded versus open eyes for each tissue and time, GSEA identified structural, signaling and metabolic pathways enriched in occluded eyes or enriched in contralateral open eyes. Similar to gene expression data, the enriched pathways in each tissue depended on sampling time. Suppl. Tables S5A and S5B list enriched pathways meeting the FDR q-value < 0.05 criterion. Shown are pathways enriched in occluded eyes relative to contralateral open eyes (generated from genes with increased expression in occluded eyes relative to open eyes) and also pathways enriched in open eyes relative to occluded eyes (generated from genes down-regulated in occluded eyes relative to open eyes). Many but not all pathways identified in retina are similar to those identified in prior genome-wide assessments of chick^22,23,25,29,44^ or mammalian^30,31,45^ myopia. Pathways related to circadian rhythms appear in each tissue.

Among the numerous and complex specific pathways, two categories will be mentioned briefly. The genes in the overall category (Table 3 and Suppl. Tables S2A, S2B) generated structural, metabolic, signaling and neural pathways in each tissue, with more identified pathways in choroid than in retina. The structural pathways found in each tissue may reflect the anatomical growth in each at myopia onset. The pathways from the occlVopen * time interaction model identified fewer pathways in retina or choroid, but they are nevertheless intriguing because the many neurotransmitter-related pathways in choroid suggest complex neural signaling in this tissue (Suppl. Tables S5A, S5B). The complexities of the GSEA-generated pathways emphasize the challenges of understanding the mechanisms initiating myopia (Suppl. Tables S5A, S5B).

We detected only a limited number of differentially expressed genes in either retina or choroid of form-deprivation myopia in chick that corresponded to candidate genes for human myopia (Table 6). Similarly, a limited number of candidate genes were identified in another strain of form-deprived chicks in a prior RNA-Seq assessment that assayed gene expression at ZT 04 on each of two separate days^22^. One possible explanation for the limited overlap in the differentially expressed chick genes and the human genes is that the mechanism of form-deprivation might differ from that of common human myopia. Confirming the utility of form-deprivation myopia, however, the anatomical alterations of form-deprivation myopia and common human myopia are similar; children develop form-deprivation myopia from conditions that degrade vision by blocking the visual axis (e.g., corneal scarring or a drooping eyelid); and, to the extent drugs can be tested in children, both experimental and human conditions respond favorably to muscarinic antagonists^9,46^. An explanation for the discrepancy could be mechanistically informative but is not now available. The pathways from the chick overall and occlVopen * time interaction categories that relate to photoreceptor biology generally conform with experimental results^47,48^ and human genetics^19,20,49^ implicating photoreceptors in refractive error development.

**Table 6:** Genes from occluded versus open chick eyes and candidate human myopia genes.

Sampling time for chick genes (ZT in hours)	Human candidate genes overlapping with differentially expressed genes in chick form deprivation myopia (occluded vs. open eye differences in chick)	
	Retina^a^	Choroid^a^
0	NOG^b^, GABRR2, BMP2^b^, ENSGALG00000011164	n.s
4	NOG^b^, PDE3A, PDE10A, L3MBTL3, PLD5, GDF11, BMP2^b^, FREM1, ENSGALG00000012847, ECEL1,	n.s
8	TSPAN10, KCNQ4, NOG^b^, GPC5, PRIMPOL, CA8, ADAMTS2, KCNMA1, ENSGALG00000053112, KCNA4, PDE3A	n.s
12	n.s	BMP3, GRM3, FGFR3^c^, SARNP, ACTC1
16	n.s	IGF-I, BMP3, ENSGALG00000052012, FREM1, DIS3L2, TMPO, KCNV2, ACTC1
20	n.s	n.s
overall	NOG^b^, GABRR2, ENSGALG00000011164, BMP2^b^	n.s

Chick genes with differences between occluded and open eyes at each sampling time (p-adj < 0.05; Suppl. Tables S2A and S2B) were compared to candidate human myopia genes identified in Karouta et al.^22^.

*n.s.* no statistically significant overlap between chick and human myopia genes.

^a^Statistically significant overlapping genes, 2-tailed Chi-square test with Yates' correction, *p* < 0.05.

^b^Also identified for chick form deprivation myopia in Karouta et al., 4-h list^22^.

^c^Also identified for chick form deprivation myopia in Karouta et al., 24-h list^22^.

Considering chick retina and choroid, distinct times of day characterized the overlap between differential gene expression in occluded versus open chick eyes and human candidate genes (Table 6). In retina, the differentially expressed chick genes corresponding to human candidate genes were identified chiefly in tissue harvested during the light phase. In choroid, the differential chick gene expression overlapping with human genes instead occurred only in tissue harvested during the dark phase (Table 6). The implication of this time-of-day difference in chick tissues for human myopia is not clear. From the perspective of circadian biology that motivated the current study, these results suggest that genes affecting human myopia could be acting at specific and different times of the day in retina and choroid.

Besides the limited overlap of involved genes in experimental versus human myopia, many other individual genes and their products have previously been identified as potential mediators of myopic eye growth in experimental animals and in children^19,20^. The sheer number of these potential mediators presents a challenge in designing studies to test hypotheses, not only in designing laboratory studies but also in translating mechanistic hypotheses to the clinic. At least in part because of the expanding list of potential genes and signaling mechanisms, approaches to myopia therapy increasingly seek non-pharmacological and behavioral mechanisms that include optical devices, behavioral modification, specific wavelengths of light exposure and outdoor activities. While generating much interest, these approaches so far have provided only modest benefits^50^.

Genetic studies in humans have implicated many genes and pathways in myopia, including circadian rhythms^19^. Recognized as a symptom of circadian disruption and supporting circadian disruption as a myopia mechanism, sleep disturbances have been identified in myopic children^51,52^. The nature of the sleep disorder differs between studies^53–56^, however, and is not consistently observed^57–59^. Studying sleep is complex and likely is impacted by environmental parameters. For instance, light pollution from artificial light at night is a serious and worsening world-wide problem^60^ that can disrupt circadian rhythms and recently has been associated with sleep disorders in children^61^. In addition, a potential causative role for myopia from exposures to light from electronic screens is generating increasing interest^62^. An influence of ambient light exposures on refractive development has long been hypothesized^18,63,64^; but the impact of light exposures, including intensity, wavelength and timing on the development of young eyes needs more direct study at both basic and clinical levels.

The current study identifies a central role for time of day in the onset of experimental myopia, buttressing a potential role for circadian biology; but a direct connection between time of day and myopia pathogenesis is now undefined. In most human studies, insufficient consideration is presently given to the nature of any potential circadian processes in clinical myopia pathogenesis or to time of day in any of the optical, behavioral or light exposures being studied as potential myopia therapies. Defining the nature of any circadian disorder underlying myopia and establishing any role for time of day in clinical myopia seem important areas to address to understand better the cause of myopia and to develop improved therapeutic interventions.

## Methods

### Animals and tissue harvesting

Newly hatched chicks (*Gallus gallus domesticus*; total number, 36 chicks; Cornell-K strain, a closed flock random-bred since the 1950’s) were reared for 12 days under a 12-h light/12-h dark cycle with ∼300 lx in cage (Phillips MAS LEDtube HF, 6500 K; https://www.lighting.philips.com/main/prof/led-lamps-and-tubes/led-tubes/master-ledtube-instantfit-hf-t8/929001284202_EU/product). At zeitgeber time (ZT) 0 (defined as lights on at ZT 0), an image-degrading diffuser was secured over the right eye using matching Velcro rings. The eye beneath an occluder is termed “occluded” and the contralateral control eye with non-impaired vision is termed “non-occluded” or “open.” Starting the next day after one full 12-h light/12-h dark cycle of device wear, chicks were killed by decapitation without anesthesia in timed cohorts so that tissues were acquired at approximately ZT 0, 4, 8, 12, 16, or 20 h (n = 6 chicks/time/condition, with chicks having been randomly assigned to time). For the “night” samples, chicks were killed under dim dark yellow light from a photographic safe light (Premier Model SL1012, Doran Manufacturing, Cincinnati OH, USA; ∼ 0.5 lx). The retina/RPE and choroid tissues were then immediately dissected separately from each eye over ice in sterile and RNAse-free conditions, snap-frozen in liquid nitrogen, shipped on dry ice to the University of Pennsylvania, and were maintained at − 80 °C until further processed. The procedure for the timing of tissue sampling is described elsewhere^24^. Based on the expression of the HINTW (histidine triad nucleotide binding protein W; ENSGALG00000035998) gene, expressed in the chicken female W chromosome, this study contained 18 female and 18 male birds overall, but the female/male numbers varied at individual times. Consequently, sex was included as a factor in the statistical model, thereby removing it as a source of variation when examining the factors of primary interest. The research was approved by the Institutional Animal Care and Use Committee of the New England College of Optometry, adhered to the ARVO Statement on the Use of Animals in Ophthalmic and Vision Research, and was performed in accordance with the relevant guidelines and regulations.

### RNA extraction and sequencing

Sample quality checks, library preparation and sequencing were performed by the Next-Generation Sequencing Laboratory (RRID:SCR_022382) in the Penn Genomics and Sequencing Core (RRID:SCR_022383). RNA was extracted from choroid and retinal tissues with the QIAGEN RNAeasy kit (QIAGEN, Germantown, MD, USA). For small pieces the entire sample was used with 300 μl of lysis buffer. For larger pieces 600 μl was used and 1/3 of the tissue was taken for library prep. Total RNA samples with a RIN (RNA Integrity Number) value of 8.9 (± 0.43) and concentrations between 22 and 672 ng/μl were produced. Library prep used the Illumina TruSeq stranded kit (Illumina, San Diego, CA, USA) with roughly equal amounts of RNA (between 100 and 300 ng). Poor quality libraries were redone. Libraries had a mean molarity of 53 nM and median insert size of 200 bp (± 42 bp). Sequencing was performed using Illumina NovaSeq 6000 (SP and S1 flow cells) to 100 bp single read sequencing. A total of 3.7 billion reads were mapped successfully to the chicken transcriptome, averaging 26.3 million mapped reads per sample.

The nature of the experiment, with a diffuser over one eye, prevented masking during sample collection. In the sequencing facility, new integer identifications were assigned to the tissue/RNA samples for library production and sequencing. Throughout, investigators had access to the sample identifications in separate lists, but sample processing included no batching that affected statistical analysis of the biological differences.

### Data analysis

Salmon^65^ was used to map reads against the transcriptome defined in Ensembl version 105.6 which was built on the chicken genome assembly GRCg6a. Two samples (one choroidal sample from an occluded eye, and a second choroidal sample from an open eye) produced insufficient reads and were eliminated. Using several Bioconductor packages in R^66^, transcriptome count data was annotated and summarized to the gene level with tximeta^67^ and further annotated with biomaRt^68^. Normalizations and statistical analyses were done using DESeq2^69^.

Normalized counts, variance stabilized counts and statistical analyses were computed with DESeq2. For time-course analyses, a reduced model was used to prioritize genes showing unequal expression across all time points. Calculations included baseMeans as the average normalized counts across all samples, *p* values, and p-adj values, the latter corresponding to the p-value corrected for the false discovery rate (FDR) using the Benjamini–Hochberg method.

Three main groups of outcome analyses were performed: (1) Gene expressions in each of the conditions (tissue, occluded eye, or open eye) were assessed separately for changes over time independent of any changes in the other conditions. Larger values of the ranking statistic provided stronger evidence for the expression of a particular gene being unequal across time points. (2) Using a conventional approach to unilateral experimental myopia, the gene expression levels in occluded eyes were compared to contralateral open control eyes at each time as the log_2_-transformed ratio of the normalized means of (occluded eyes) versus (contralateral open eyes) with p-adj values for statistical significance at each time. (3) To identify those genes with varying expression in the occluded eye different from the variation pattern of the contralateral open eye, we modeled statistically the interaction between treatment (occluded vs. open eye) and time (i.e., occlVopen * time) for each tissue. Venn diagrams (http://www.interactivenn.net/index.html)^28^ were used to identify differentially expressed genes at more than on time during the day.

Likelihood ratio tests (LRT) were calculated by DESeq2 statistics^69^ with a reduced model to prioritize genes that showed unequal expression across all time points. Genes that were significantly changed across the timeline were clustered into groups sharing similar expression patterns and displayed in graphs (Figs. 1, 2) with the degPatterns function from the DEGreport package^70^. Clusters are depicted with group names and orders assigned by the degPatterns function. Statistical results for pairwise comparisons between occluded and contralateral open eyes were examined for pathway enrichment with Gene Set Enrichment Analysis (GSEA; v4.2.3)^71^. Using the totality of genes studied, GSEA assesses statistically whether an a priori set of genes associates with particular phenotypes based on the relative enrichment or reduction of sets of genes. Enrichment analyses were done in pre-ranked mode using the DESeq2 statistic as the ranking metric, and tested against the Canonical Pathways collection (C2:CP) of the Molecular Signatures Database (MSigDB; v7.5.1; https://www.gsea-msigdb.org/gsea/msigdb). We chose the C2:CP (canonical pathways) curated gene set in the Human Molecular Signatures Database because, based on preliminary analysis, its pathway classifications provided useful pathways (e.g., structure, inflammation, neurotransmission, peptide signaling, photoreception, etc.) to generate hypotheses for myopia pathogenesis.

To compare identified chicken genes to a recently published list of human genes associated with clinical myopia^22^, the chicken transcriptome (as Ensembl gene identifications) was mapped via orthologs to the human gene symbols. The genes with statistically significant likelihood ratios of the occluded-versus-open eye assessments and genes with the statistically significant likelihood ratios of the treatment-time interactions of chick were compared to the human gene list using a Chi-square test with Yates' correction. Unless otherwise specified, a value of p-adj or FDR less than 0.05 was considered statistically significant. The study is reported in accordance with the ARRIVE guidelines.

### Supplementary Information

Supplementary Legends.

Supplementary Figure S1A.

Supplementary Figure S1B.

Supplementary Table S1.

Supplementary Table S2A.

Supplementary Table S2B.

Supplementary Table S3.

Supplementary Table S4A.

Supplementary Table S4B.

Supplementary Table S5A.

Supplementary Table S5B.

Supplementary Table S6A.

Supplementary Table S6B.

## Data Availability

The data generated in this study were deposited in GEO (Gene Expression Omnibus) with accession number GSE227724 (https://www.ncbi.nlm.nih.gov/geo/query/acc.cgi?acc=GSE227724).
