# Supplementary material for: Diurnal retinal and choroidal gene expression patterns support a role for circadian biology in myopia pathogenesis
Source: Sci Rep. 2024 Jan 4;14:533. doi: 10.1038/s41598-023-50684-2 (PMC10767138; doi:10.1038/s41598-023-50684-2)
Supplement: Supplementary file 1 — Supplementary Legends. [file 41598_2023_50684_MOESM1_ESM.docx]

**Supplementary Materials**

**Table of Contents and Legends**

**Suppl. Figure S1A. Retinal heatmap: occluded vs. open eye differences interacting with time.**

Heatmap of the log_2_ fold-changes in retina are shown across the sampling times for the occluded vs. open eye differences interacting with time.

Key in upper right gives the magnitude of log_2_ fold-changes, and the color shows the direction of gene expression differences between occluded and open eyes – red, expression higher in occluded eye; blue, expression lower in the occluded eye.

*lfc;* log_2_ fold-change, followed by the ZT of the tissue sampling time.

Left ordinate: dendrogram indicating the arrangements of the gene clusters.

Right ordinate: gene name.

See Figure 2, Table 4, and Suppl. Table 3A.

**Suppl. Figure S1B. Choroidal heatmap: occluded vs. open eye differences interacting with time.**

Heatmap of the log_2_ fold-changes in choroid are shown across the sampling times for the occluded vs. open eye differences interacting with time.

Key in upper right gives the magnitude of log_2_ fold-changes, and the color shows the direction of gene expression differences between occluded and open eyes – red, expression higher in occluded eye; blue, expression lower in the occluded eye.

*lfc:* log_2_ fold-change, followed by the ZT of the tissue sampling time.

Left ordinate: dendrogram indicating the arrangements of the gene clusters.

Right ordinate: gene name.

See Figure 2, Table 4, and Suppl. Table 3B.

**Suppl. Table S1.** **Gene expression variability over time.**

The variability over time for genes with measurable p-adj values that vary over time are shown for A) retinas of occluded eyes; B) retinas of contralateral open eyes; C) choroids of occluded eyes; and D); choroids of contralateral open eyes.

*baseMean,* average normalized counts across all samples.

*p-adj,* p-value corrected for the false discovery rate (FDR) using the Benjamini-Hochberg method.

**Suppl. Table S2. Occluded vs. open eye comparisons.**

The gene expression levels in occluded vs. contralateral open eyes are compared over time. Genes with inter-eye differences with p-adj<0.05 are listed and sorted by log2 fold-change: A) retina; and B) choroid.

*ZT,* Zeitgeber time of tissue sampling, in hours.

*overall,* genes identified by the considering all replicates at all time points simultaneously (see text, Table 2).

*lfcSE,* standard error of the log2 fold-change.

*p-adj,* p-value corrected for the false discovery rate (FDR) using the Benjamini-Hochberg method.

**Suppl. Table S3. Group assignments of inter-eye expression patterns interacting with time.**

The inter-eye gene differences that interacted with time, by the criterion of p-adj<0.1, are assigned to the groups illustrated in Figure 2: A) retina; and B) choroid. The assignments are sorted by group.

*p-adj,* p-value corrected for the false discovery rate (FDR) using the Benjamini-Hochberg method.

*group,* see Figure 3 in text for the interaction patterns with time of the gene expression changes in the occluded vs. open eyes.

*not assig.,* a gene meeting the statistical criterion of p-adj<0.1 for the inter-eye difference interacting with time but not assignable into a modeled group.

**Suppl. Table S4. Gene expression differences in occluded vs. open eyes at more than one time.**

Derived from the Venn Diagrams of Figure 3 with inter-eye expression differences of p-adj< 0.05, genes differentially expressed a more than one ZT time, the number of genes, the gene names/descriptions, and the directions of gene expression changes for each set of genes are shown. A) retina; B) choroid.

*ZT,* Zeitgeber time of tissue sampling, in hours.

**Suppl. Table S5. GSEA Pathways, all times.**

Pathways were assigned for gene expression differences in occluded vs. open eyes. Based on GSEA, two general types of pathways were identified: those enriched in occluded eyes relative to the contralateral open control eyes generated from genes with increased expression in occluded eyes relative to open eyes; and those enriched in open eyes which correspond to genes with decreased expression in occluded eyes relative to contralateral open eyes. The pathways include those varying over time during the 24-hour day, the “overall” category (see Suppl. Tables 2A and 2B), and the pathways for the interaction of occluded vs. open eye differences and time (i.e., occlVopen*time interaction; see Table 4, Suppl. Table S3) for each tissue. Pathways listed met a broad range of statistical significance up to FDR q-value<0.05. A) retina; B) choroid.

*GSEA,* Gene Set Enrichment Analysis

*ZT,* Zeitgeber time of tissue sampling, in hours.

*NES,* normalized enrichment score

*FDR,* false discovery rate

**Suppl. Table S6. Complete data on occluded vs. open eye comparisons.**

Gene expression levels in occluded vs. contralateral open eyes over time are compared for all genes. Listed are all genes with log2 fold-changes, sorted by p-adj values. A) retina; and B) choroid.

*ZT,* Zeitgeber time of tissue sampling, in hours.

*overall,* genes identified by the considering all replicates at all time points simultaneously (see text, Table 2).

*baseMean,* average normalized counts across all samples.

*lfcSE,* standard error of the log_2_ fold-change.

*p-adj,* p-value corrected for the false discovery rate (FDR) using the Benjamini-Hochberg method.
