# Supplementary material for: Diurnal retinal and choroidal gene expression patterns support a role for circadian biology in myopia pathogenesis
Source: Sci Rep. 2024 Jan 4;14:533. doi: 10.1038/s41598-023-50684-2 (PMC10767138; doi:10.1038/s41598-023-50684-2)
Supplement: Supplementary file 3 — Supplementary Figure S1B. [file 41598_2023_50684_MOESM3_ESM.docx]

| **Supplementary Figure S1B. Choroidal heatmap: occluded vs. open eye differences interacting with time.** |
| --- |
| 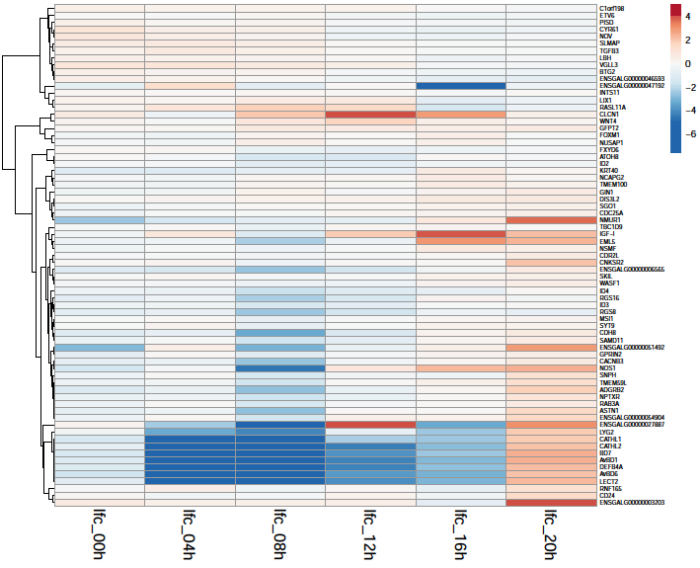 |
| Heatmap of the log_2_ fold-changes in choroid are shown across the sampling times for the occluded vs. open eye differences interacting with time.  Key in upper right gives the magnitude of log_2_ fold-changes, and the color shows the direction of gene expression differences between occluded and open eyes – red, expression higher in occluded eye; blue, expression lower in the occluded eye. See Figure 2, Table 4, and Suppl. Table S3B.  *lfc:* log_2_ fold-change, followed by the ZT of the tissue sampling time.  Left ordinate: dendrogram indicating the arrangements of the gene clusters.  Right ordinate: gene name. |
