# Supplementary material for: Diurnal retinal and choroidal gene expression patterns support a role for circadian biology in myopia pathogenesis
Source: Sci Rep. 2024 Jan 4;14:533. doi: 10.1038/s41598-023-50684-2 (PMC10767138; doi:10.1038/s41598-023-50684-2)
Supplement: Supplementary file 8 — Supplementary Table S4A. [file 41598_2023_50684_MOESM8_ESM.docx]

**Supplementary Table S4A. Retinal genes differentially expressed in occluded vs. open eyes at more than one time, p-adj<0.05.**

| **Genes differentially expressed at more than one ZT time** | | **Gene**  **Name** | **Gene description** | **Direction of gene expression change at common times** | | | | | | | | |
| --- | --- | --- | --- | --- | --- | --- | --- | --- | --- | --- | --- | --- |
| **ZT times of tissue sampling (hour)** | **Number of genes** |  |  |  |  |  |  |  |  |  |  |  |
|  |  |  |  |  | **ZT time and log2 FoldChange** | | | | | | | |
|  |  |  |  |  | 0 hr | 4 hr | 8 hr | 12 hr | | 16 hr | | 20 hr |
| 0 & 4 | 2 | BMP2 | bone morphogenetic protein 2 | all decrease | -2.05 | -1.25 |  |  | |  | |  |
|  |  | G0S2 | G0/G1 switch 2 | all increase | 0.71 | 0.59 |  |  | |  | |  |
| 0 & 8 | 2 | ARID5B | AT-rich interaction domain 5B | all decrease | -0.36 |  | -0.23 |  | |  | |  |
|  |  | LONRF3 | LON peptidase N-terminal domain and ring finger 3 | all decrease | -0.44 |  | -0.40 |  | |  | |  |
| 4 & 8 | 8 | SPON1 | spondin 1 | all decrease |  | -0.37 | -0.33 |  | |  | |  |
|  |  | UNC5C | unc-5 netrin receptor C | all decrease |  | -0.37 | -0.32 |  | |  | |  |
|  |  | PDE3A | phosphodiesterase 3A | all decrease |  | -0.40 | -0.41 |  | |  | |  |
|  |  | ENSGALG00000031866 | protein NDNF-like | all increase |  | 0.36 | 0.30 |  | |  | |  |
|  |  | GRB10 | growth factor receptor bound protein 10 | all decrease |  | -0.36 | -0.35 |  | |  | |  |
|  |  | ENSGALG00000010854 | histone deacetylase 9 | all decrease |  | -0.21 | -0.31 |  | |  | |  |
|  |  | RSPO2 | R-spondin 2 | all decrease |  | -0.77 | -0.78 |  | |  | |  |
|  |  | ALKAL2 | family with sequence similarity 150 member B | all decrease |  | -0.90 | -0.84 |  | |  | |  |
| 4 & 12 | 1 | CRHBP | corticotropin releasing hormone binding protein | all decrease |  | -3.73 |  | -3.32 | |  | |  |
| 8 & 12 | 7 | ENSGALG00000007803 | solute carrier family 2, facilitated glucose transporter member 9-like | all increase |  |  | 2.13 | | 1.36 | |  |  |
|  |  | MAFF | MAF bZIP transcription factor F | all decrease |  |  | -0.58 | | -0.40 | |  |  |
|  |  | TH | tyrosine hydroxylase | all decrease |  |  | -1.19 | | -1.64 | |  |  |
|  |  | MMP9 | matrix metallopeptidase 9 | all decrease |  |  | -0.70 | | -0.89 | |  |  |
|  |  | CREB3L1 | cAMP responsive element binding protein 3 like 1 | all decrease |  |  | -0.69 | | -0.63 | |  |  |
|  |  | NTS | neurotensin | all increase |  |  | 0.42 | | 0.51 | |  |  |
|  |  | GCG | glucagon | all decrease |  |  | -0.53 | | -0.59 | |  |  |
| 8 & 16 | 1 | MXI1 | MAX interactor 1, dimerization protein | all decrease |  |  | -0.19 | |  | | -0.23 |  |
| 0 & 4 & 8 & 12 | 3 | UTS2B | urotensin 2B | all decrease | -1.10 | -0.95 | -1.34 | | -1.35 | |  |  |
|  |  | DUSP4 | dual specificity phosphatase 4 | all decrease | -0.94 | -0.60 | -0.90 | | -0.89 | |  |  |
|  |  | NOG | noggin | all increase | 0.82 | 0.55 | 1.04 | | 0.61 | |  |  |
| 0 & 8 & 12 | 2 | PCSK1 | proprotein convertase subtilisin/kexin type 1 | all decrease | -0.65 |  | -0.40 | | -0.35 | |  |  |
|  |  | ENSGALG00000005011 | SHC adaptor protein 4 | all decrease | -0.64 |  | -0.55 | | -0.49 | |  |  |
| 4 & 8 & 12 | 3 | DIO2 | deiodinase, iodothyronine type II | all decrease |  | -0.67 | -0.56 | | -0.48 | |  |  |
|  |  | GLS2 | glutaminase 2 | all decrease |  | -0.24 | -0.38 | | -0.32 | |  |  |
|  |  | VIP | vasoactive intestinal peptide | all decrease |  | -0.68 | -1.47 | | -1.41 | |  |  |
| 8 & 12 & 16 | 1 | GAD2 | glutamate decarboxylase 2 | all decrease |  |  | -0.37 | | -0.32 | | -0.34 |  |
|  |  |  |  |  |  |  |  | |  | |  |  |
