# Supplementary material for: Diurnal retinal and choroidal gene expression patterns support a role for circadian biology in myopia pathogenesis
Source: Sci Rep. 2024 Jan 4;14:533. doi: 10.1038/s41598-023-50684-2 (PMC10767138; doi:10.1038/s41598-023-50684-2)
Supplement: Supplementary file 9 — Supplementary Table S4B. [file 41598_2023_50684_MOESM9_ESM.docx]

**Supplementary Table S4B. Choroidal genes differentially expressed in occluded vs. open eyes at more than one time, p-adj<0.05.**

| **Genes differentially expressed at more than one ZT time** | | **Gene Name** | **Gene description** | **Direction of gene expression change** | | | | | | |
| --- | --- | --- | --- | --- | --- | --- | --- | --- | --- | --- |
| **ZT times** **of tissue sampling**  **(hour)** | **Number of genes** |  |  |  |  |  |  |  |  |  |
|  |  |  |  |  | **ZT time and log2 FoldChange** | | | | | |
|  |  |  |  |  | 0 | 4 | 8 | 12 | 16 | 20 |
| 0 & 8 | 2 | NGF | nerve growth factor | all increase | 0.78 |  | 0.46 |  |  |  |
|  |  | C1orf198 | chromosome 3 open reading frame, human C1orf198 | all increase | 0.45 |  | 0.37 |  |  |  |
| 0 & 12 | 1 | MYL3 | myosin, light chain 3, alkali; ventricular, skeletal, slow | all decrease | -8.09 |  |  | -17.49 |  |  |
| 0 & 16 | 1 | HIST1H2B7 | histone cluster 1, H2B-VII (similar to human histone cluster 1, class H2B, member N) | all decrease | -18.81 |  |  |  | -50.30 |  |
| 0 & 20 | 2 | KRT7 | keratin 7 | all increase | 1.27 |  |  |  |  | 1.19 |
|  |  | ENSGALG00000029842 | solute carrier organic anion transporter family member 5A1 | all increase | 0.87 |  |  |  |  | 1.06 |
| 0 & 8 & 12 | 1 | PPP4R4 | protein phosphatase 4 regulatory subunit 4 | all increase | 0.74 |  | 0.70 | 0.79 |  |  |
| 0 & 8 & 16 | 1 | NELL2 | neural EGFL like 2 |  | -0.90 |  | -0.93 |  | --1.02 |  |
| 0 & 8 & 20 | 2 | PDGFD | platelet derived growth factor D | all increase | 0.46 |  | 0.47 |  |  | 0.45 |
|  |  | HAS2 | hyaluronan synthase 2 |  | -1.70 |  | -0.63 |  |  | -1.53 |
| 0 & 4 & 8 | 3 | GJC2 | gap junction protein gamma 2 | all increase | 0.86 | 0.72 | 0.61 |  |  |  |
|  |  | SLMAP | sarcolemma associated protein | all increase | 0.63 | 0.77 | 0.51 |  |  |  |
|  |  | VGLL3 | vestigial like family member 3 | all increase | 0.94 | 1.00 | 0.72 |  |  |  |
| 0 & 4 & 8 & 12 & 20 | 2 | CER1 | cerberus 1, DAN family BMP antagonist | all decrease | -2.32 | -1.66 | -2.19 | -1.51 |  | -2.21 |
|  |  | GRM3 | glutamate metabotropic receptor 3 | all increase | 1.19 | 1.12 | 1.18 | 0.90 |  | 1.04 |
| 0 & 4 & 8 & 16 & 20 | 1 | ENSGALG00000017029 | fibrinogen-like protein 1-like | all decrease | -2.85 | -1.97 | -2.47 |  | -2.71 | -2.99 |
| 0 & 8 & 12 & 16 | 2 | C1QTNF7 | C1q and TNF related 7 | all increase | 0.90 |  | 0.90 | 0.96 | 0.80 |  |
|  |  | BMP3 | bone morphogenetic protein 3 | all increase | 1.22 |  | 1.62 | 1.87 | 1.21 |  |
| 0 & 8 & 12 & 20 | 2 | ASB2 | ankyrin repeat and SOCS box containing 2 | all increase | 0.98 |  | 1.21 | 1.33 |  | 0.87 |
|  |  | PTX3 | pentraxin 3 | all decrease | -2.00 |  | -2.28 | -1.98 |  | -2.02 |
| 0 & 8 & 12 & 16 & 20 | 2 | CORIN | corin, serine peptidase | all increase | 1.69 |  | 1.51 | 1.37 | 1.54 | 1.68 |
|  |  | PTHLH | parathyroid hormone like hormone | all increase | 1.74 |  | 2.27 | 1.25 | 1.68 | 1.25 |
| 0 & 8 & 16 & 20 | 2 | FAM26E | family with sequence similarity 26 member E | all increase | 1.53 |  | 1.13 |  | 1.67 | 1.57 |
|  |  | HTR1B | 5-hydroxytryptamine receptor 1B | all decrease | -0.93 |  | -1.27 |  | -1.06 | -0.93 |
| 4 & 8 | 11 | TGFB3 | transforming growth factor beta 3 | all increase |  | 0.72 | 0.46 |  |  |  |
|  |  | CATHL2 | cathelicidin antimicrobial peptide | all decrease |  | -6.17 | -6.78 |  |  |  |
|  |  | DEFB4A | defensin beta 4A | all decrease |  | -5.62 | -6.25 |  |  |  |
|  |  | CATHL1 | cathelicidin-1 | all decrease |  | -4.94 | -5.96 |  |  |  |
|  |  | BD7 | avian beta-defensin 7 | all decrease |  | -5.70 | -6.02 |  |  |  |
|  |  | AvBD1 | avian beta-defensin 1 | all decrease |  | -5.91 | -6.41 |  |  |  |
|  |  | LECT2 | leukocyte cell derived chemotaxin 2 | all decrease |  | -5.38 | -6.91 |  |  |  |
|  |  | AvBD4 | avian beta-defensin 4 | all decrease |  | -7.38 | -6.34 |  |  |  |
|  |  | AvBD6 | avian beta-defensin 6 | all decrease |  | -5.78 | -7.64 |  |  |  |
|  |  | SEPT5 | septin 5 | all increase |  | 0.50 | 0.36 |  |  |  |
|  |  | ENSGALG00000004804 | transglutaminase 3 | all decrease |  | -6.00 | -7.50 |  |  |  |
| 4 & 8 & 12 & 20 | 1 | DEPDC1 | DEP domain containing 1 | all decrease |  | -0.78 | -0.90 | -1.10 |  | -0.69 |
| 4 & 12 | 2 | ENSGALG00000048153 |  | all decrease |  | -18.3 |  | -20.4 |  |  |
|  |  | ENSGALG00000046148 |  | decrease ZT4; increase ZT12 |  | -18.5 |  | 16.5 |  |  |
| 4 & 20 | 2 | ENSGALG00000050918 |  | all decrease |  | -16.01 |  |  |  | -15.93 |
|  |  | ENSGALG00000050826 |  | all decrease |  | -16.38 |  |  |  | -23.68 |
| 8 & 12 | 19 | ID3 | inhibitor of DNA binding 3, HLH protein | all decrease |  |  | -1.39 | -0.86 |  |  |
|  |  | RGS16 | regulator of G-protein signaling 16 | all decrease |  |  | -2.99 | -1.36 |  |  |
|  |  | RGS8 | regulator of G-protein signaling 8 | all decrease |  |  | -2.43 | -1.51 |  |  |
|  |  | ATOH8 | atonal bHLH transcription factor 8 | all decrease |  |  | -1.37 | -1.26 |  |  |
|  |  | ID2 | inhibitor of DNA binding 2, HLH protein | all decrease |  |  | -0.90 | -0.89 |  |  |
|  |  | STC2 | stanniocalcin 2 | all decrease |  |  | -1.94 | -1.79 |  |  |
|  |  | ENSGALG00000047567 |  | all decrease |  |  | -1.38 | -1.20 |  |  |
|  |  | TBC1D8 | TBC1 domain family member 8 | all increase |  |  | 0.60 | 0.58 |  |  |
|  |  | LMO1 | LIM domain only 1 (rhombotin 1) | all decrease |  |  | -0.61 | -0.62 |  |  |
|  |  | FXYD6 | FXYD domain containing ion transport regulator 6 | all decrease |  |  | -0.52 | -0.61 |  |  |
|  |  | FHL3 | four and a half LIM domains 3 | all increase |  |  | 0.40 | 0.44 |  |  |
|  |  | PGR2/3 | tandem PRG2/PRG3 gene pair | all decrease |  |  | -5.93 | -20.92 |  |  |
|  |  | NOG2 | noggin 2 | all decrease |  |  | -1.90 | -2.35 |  |  |
|  |  | SARNP | SAP domain containing ribonucleoprotein | all decrease |  |  | -0.42 | -0.62 |  |  |
|  |  | ENSGALG00000043352 | ceramide synthase 4 | all increase |  |  | 0.41 | 0.55 |  |  |
|  |  | SPSB1 | splA/ryanodine receptor domain and SOCS box containing 1 | all increase |  |  | 0.41 | 0.50 |  |  |
|  |  | MAPRE2 | microtubule associated protein RP/EB family member 2 | all increase |  |  | 0.33 | 0.38 |  |  |
|  |  | LRIG1 | leucine rich repeats and immunoglobulin like domains 1 | all increase |  |  | 0.86 | 1.06 |  |  |
|  |  | WNT4 | Wnt family member 4 | all increase |  |  | 0.91 | 1.78 |  |  |
| 8 & 20 | 7 | CIBAR1 | family with sequence similarity 92 member A | all increase |  |  | 0.72 |  |  | 0.71 |
|  |  | PROK2 | prokineticin 2 | all decrease |  |  | -2.77 |  |  | -2.43 |
|  |  | MCC | mutated in colorectal cancers | all increase |  |  | 0.47 |  |  | 0.56 |
|  |  | PGR | progesterone receptor | all increase |  |  | 0.98 |  |  | 1.15 |
|  |  | NPNT | nephronectin | all increase |  |  | 0.62 |  |  | 0.92 |
|  |  | ENSGALG00000036838 | peroxidasin like | decrease ZT8; increase ZT20 |  |  | -3.40 |  |  | 3.94 |
|  |  | PKP2 | plakophilin 2 | all increase |  |  | 0.51 |  |  | 0.62 |
| 8 & 12 & 16 & 20 | 1 | GFPT2 | glutamine-fructose-6-phosphate transaminase 2 | all increase |  |  | 0.76 | 0.69 | 0.57 | 0.63 |
| 8 & 12 & 16 | 1 | GDPD4 | glycerophosphodiester phosphodiesterase domain containing 4 | all decrease |  |  | -0.77 | -0.84 | -0.67 |  |
| 8 & 12 & 20 | 1 | MXRA8 | matrix remodeling associated 8 | all increase |  |  | 0.76 | 0.64 |  | 0.49 |
| 8 & 16 | 5 | TMEM47 | TNF receptor superfamily member 19 | all increase |  |  | 0.64 |  | 0.66 |  |
|  |  | OLFML1 | olfactomedin like 1 | all increase |  |  | 0.91 |  | 0.75 |  |
|  |  | MICAL2 | microtubule associated monooxygenase, calponin and LIM domain containing 2 | all increase |  |  | 0.63 |  | 0.72 |  |
|  |  | THY1 | Thy-1 cell surface antigen | all increase |  |  | 0.62 |  | 0.72 |  |
|  |  | MCM6 | minichromosome maintenance complex component 6 | all increase |  |  | 0.25 |  | 0.37 |  |
| 8 & 16 & 20 | 2 | CYGB | cytoglobin | all increase |  |  | 0.53 |  | 0.73 | 0.63 |
|  |  | HTRA3 | HtrA serine peptidase 3 | all increase |  |  | 0.58 |  | 0.97 | 0.93 |
| 12 & 16 | 2 | CLCN1 | chloride voltage-gated channel 1 | all increase |  |  |  | 4.14 | 2.99 |  |
|  |  | TNFRSF19 | TNF receptor superfamily member 19 | all increase |  |  |  | 0.84 | 0.75 |  |
| 16 & 20 | 11 | ENSGALG00000052368 |  | increase ZT16; decrease ZT20 |  |  |  |  | 16.94 | -21.99 |
|  |  | DIS3L2 | DIS3 like 3'-5' exoribonuclease 2 | all increase |  |  |  |  | 0.76 | 0.80 |
|  |  | DSP | desmoplakin | all increase |  |  |  |  | 2.23 | 1.47 |
|  |  | ENSGALG00000054035 |  | all increase |  |  |  |  | 18.61 | 19.68 |
|  |  | TFDP2 | transcription factor Dp-2 | all increase |  |  |  |  | 0.57 | 0.66 |
|  |  | OXSR1 | oxidative stress responsive 1 | all increase |  |  |  |  | 0.73 | 0.65 |
|  |  | ENSGALG00000003283 | cartilage oligomeric matrix protein | all increase |  |  |  |  | 1.19 | 1.38 |
|  |  | ENSGALG00000052522 |  | increase ZT16; decrease ZT20 |  |  |  |  | 27.36 | -38.80 |
|  |  | SKIL | SKI like proto-oncogene | all increase |  |  |  |  | 0.39 | 0.50 |
|  |  | FREM1 | FRAS1 related extracellular matrix 1 | all increase |  |  |  |  | 1.10 | 1.18 |
|  |  | PSPC1 | paraspeckle component 1 | all decrease |  |  |  |  | -0.35 | -0.45 |
